# The dose–response effects of hydrolyzed curcumin on recovery, inflammation, and oxidative stress following exercise‐induced muscle damage in males

**DOI:** 10.14814/phy2.70504

**Published:** 2025-08-09

**Authors:** Toby A. Helder, Sean Condon, Ian Grant, Nathan A. Lewis, Jessica Hill, Charles R. Pedlar, Luke Hughes

**Affiliations:** ^1^ Faculty of Sport, Technology and Health Sciences St. Mary's University Twickenham London UK; ^2^ Orreco, Business Innovation Unit University of Galway Galway Ireland; ^3^ Applied and Human Sciences Kingston University London UK; ^4^ UK Sports Institute, Athletes Training Village University of Bath Bath UK; ^5^ Institute of Sport, Exercise and Health, Division of Surgery and Interventional Science University College London London UK; ^6^ Department of Sport Exercise & Rehabilitation Northumbria University Newcastle upon Tyne UK

**Keywords:** antioxidants, delayed onset muscle soreness, eccentric exercise, redox, turmeric

## Abstract

This study investigated the effects of two dosages (750 mg and 1500 mg) of hydrolyzed curcumin on physiological recovery following exercise‐induced muscle damage (EIMD). In a randomized, placebo‐controlled, double‐blind design, 34 recreationally active males (27 ± 6 years; 180 ± 7.3 cm; 82 ± 11.3 kg) were assigned to three groups: PLA (2 × 750‐mg/day placebo), LOW (1 × 750‐mg/day curcumin + 1 × 750‐mg/day placebo), and HIGH (2 × 750‐mg/day curcumin). Supplements were delivered in 15 mL gel sachets over 7 days, starting 48 h before EIMD. The EIMD protocol involved 8 sets of 10 repetitions at 110% of one‐repetition maximum on the leg press, with 5‐s eccentric phases and assisted concentric phases, targeting the quadriceps. Recovery was assessed pre, post, 24, 48, and 72 h post‐EIMD via the Free Oxygen Radical Test (FORT), creatine kinase (CK), interleukin‐6 (IL‐6), isokinetic peak power, and a muscle endurance test (sustained isometric contraction at 50% peak torque). The HIGH group showed significantly greater reductions in pain, CK, FORT, and IL‐6 (*p* < 0.05), but slower muscle endurance recovery at 24 h compared to LOW. Findings suggest a dose–response effect, with higher curcumin doses improving biochemical recovery but potentially impairing performance recovery.

## 1 INTRODUCTION

High intensity physical exercise can lead to exercise‐induced muscle damage (EIMD) and delayed onset muscle soreness (DOMS) (Byrne et al., 2004). As a consequence of mechanical loading, sarcomeres lengthen unevenly under tension, exhibiting nonuniform stretching until they extend beyond the point of myofilament overlap. Inevitably, the myofilament structures become overstretched and unable to overlap within the sarcomere (Owens et al., 2019). The structural damage that occurs during the primary phase triggers a secondary inflammatory response (Miles et al., 2008) which produces reactive oxygen species (ROS). ROS are essential for redox signaling pathways for adaptation (Lennicke & Cochemé, 2021). However, if uncontrolled, ROS may cause further muscle damage, slowing down recovery (Peake et al., 2017; Powers & Jackson, 2008). This can increase localized oedema, induce DOMS, decrease the ability to generate muscle force, and increase the presence of muscle proteins such as creatine kinase (CK) in the blood (Fatouros & Jamurtas, 2016). The impairment of muscle function resulting from EIMD and DOMS can affect subsequent short‐term athletic performance as well as render an individual more susceptible to injury (Beba et al., 2022). Minimizing the negative effects of inflammatory and oxidative responses may improve recovery and prevent injury (Park & Kwak, 2016; Slattery et al., 2015). This could be particularly beneficial during competitive seasons in sports such as football, as it may reduce muscle fatigue from repeated training and competition exposure (Margaritelis et al., 2020). Consequently, strategies capable of controlling oxidative and inflammatory responses and minimizing muscle damage have been progressively researched (Basham et al., 2020; Bell et al., 2016; Choi et al., 2023).

Nonsteroidal anti‐inflammatory drugs (NSAIDs) are utilized to help ease the inflammatory response from DOMS (Myburgh, 2014; Qamar et al., 2019; Schoenfeld, 2012). However, long‐term use of such drugs may inhibit adaptation by inhibiting the initial stages of healing of the cyclooxygenase (COX) 1 and 2 pathways and reduction in Prostaglandin 2 (PG2) production (Auriel et al., 2014). Polyphenol supplements such as curcumin may offer a substitute strategy to this problem (Schiborr et al., 2014). Curcumin is a highly pleiotropic molecule that has been found to interact with multiple anti‐inflammatory (Shehzad & Lee, 2013) and antioxidant pathways (Kocaadam & Şanlier, 2017). Interest in curcumin has grown due to its potential to act through mechanisms similar to NSAIDs, though with less pronounced anti‐inflammatory effects (Mallard et al., 2021; McFarlin et al., 2016). Curcumin may significantly influence various inflammation regulators, leading to the inhibition of transcription factor Nuclear Factor Kappa‐B (NF‐kB) activation, a reduction in Activator Protein‐1 (AP‐1) binding to DNA, and suppression of the COX‐2 pathway (Liu et al., 2024). For a detailed review of the molecular targets of curcumin see Liu et al. (2024).

Furthermore, positive effects on inflammatory cytokines have been observed when curcumin is consumed prior to exercise (McFarlin et al., 2016; Tanabe et al., 2019). Nevertheless, there are few data regarding what might constitute the ideal dose of curcumin on recovery markers post muscle damage. A range of doses has been studied, ranging from 150 mg/day to 2000 mg/day (Basham et al., 2020). The minimum effective dose and the ideal dosage for achieving maximum benefits from curcumin have not been established in studies. However, the effect of curcumin on reducing tumor necrosis factor‐α (TNF‐α) was not different when taking 150 mg/day (Tanabe et al., 2015) or 180 mg/day (Tanabe et al., 2019). Moreover, McFarlin et al. (2016) showed the consumption of 400 mg/day of curcumin, both immediately preceding and up to 4 days post EIMD, led to a reduction in muscle damage (CK) and inflammation (TNF‐α) by 25%–47% compared to a placebo. Additionally, Nicol et al. (2015) reported that a curcumin dose of 500 mg/day significantly mitigated muscle soreness, while McFarlin et al. (2016) observed no discernible difference with a dose of 400 mg/day. This suggests there may be a dose–response with larger dosages of ≥400 mg/day (Basham et al., 2020; Drobnic et al., 2014) though an optimum dose is yet to be established. One explanation could be the low bioavailability of natural curcumin, leading to relatively low plasma concentrations despite high oral intake, thereby limiting its impact (Obrenovich et al., 2022). To combat the poor absorption of curcumin, which is largely due to its water insolubility and poor metabolism in the small intestine, studies have investigated different curcumin formulations to improve plasma levels (Heger et al., 2014). One method that is yet to be investigated with recovery is curcumin in a hydrolyzed (liposomal) form. This formulation increases curcumin bioavailability through enhancing its water solubility, thus increasing plasma curcumin (Schiborr et al., 2014).

The aim of this study was to examine the effect of two doses (750 and 1500 mg/day) of hydrolyzed curcumin on physiological recovery following EIMD, through the assessment of indirect and direct markers of muscle function, damage, inflammation, and oxidative stress. We hypothesized that: (a) participants in both curcumin groups would exhibit enhanced functional recovery and reduced perceived muscle soreness compared to placebo; (b) pro‐inflammatory and oxidative stress markers would be lower in both curcumin groups compared to the placebo group; and (c) the 1500 mg curcumin dose would provoke greater effects than the 750 mg dose.

## 2 MATERIALS AND METHODS

### 2.1 Participants

Thirty‐four recreationally active male participants (age range 18–41 years) volunteered for the study. All had similar training histories and were familiar with the leg press machine; see Table 1 for participant characteristics. Participants were asked to refrain from exercise, alcohol, and pharmacological substances/supplements for 48 h prior to and during the protocol. Caffeine consumption was ceased 12 h before each trial. Participants confirmed they did not consume turmeric regularly, either as a supplement or through their diet, and were instructed to discontinue dietary or supplementary turmeric consumption in the week leading up to the trial through to the end of the experimental protocol while otherwise maintaining their usual dietary habits throughout the study. Participants were excluded if they had sustained an injury within the previous 6 months. Ethics approval was granted by the University Ethics Committee (SMU_ETHICS_2021‐22_218). All procedures were conducted in accordance with the Declaration of Helsinki. Written informed consent was obtained from all participants. Upon study completion, one participant exhibited implausible biomarker values and was therefore excluded from analysis, reducing the LOW group's sample size to *n* = 11.

**TABLE 1:** Participant characteristics for the placebo group (PLA), single dose group (LOW), and double dose group (HIGH) (Mean ± SD).

Group	Age (y)	Height (cm)	Weight (kg)	1RM leg press (kg)
PLA (*n* = 10)	27 ± 6	181.9 ± 8.6	84.2 ± 13.1	232 ± 49.4
LOW (*n* = 11)	27 ± 6	178.37 ± 5.8	83.9 ± 9.2	268 ± 55.4
HIGH (*n* = 12)	27 ± 7	179 ± 8	81 ± 12.9	272.3 ± 7

Abbreviation: 1RM, one repetition maximum.

### 2.2 Experimental overview

**FIGURE 1:**
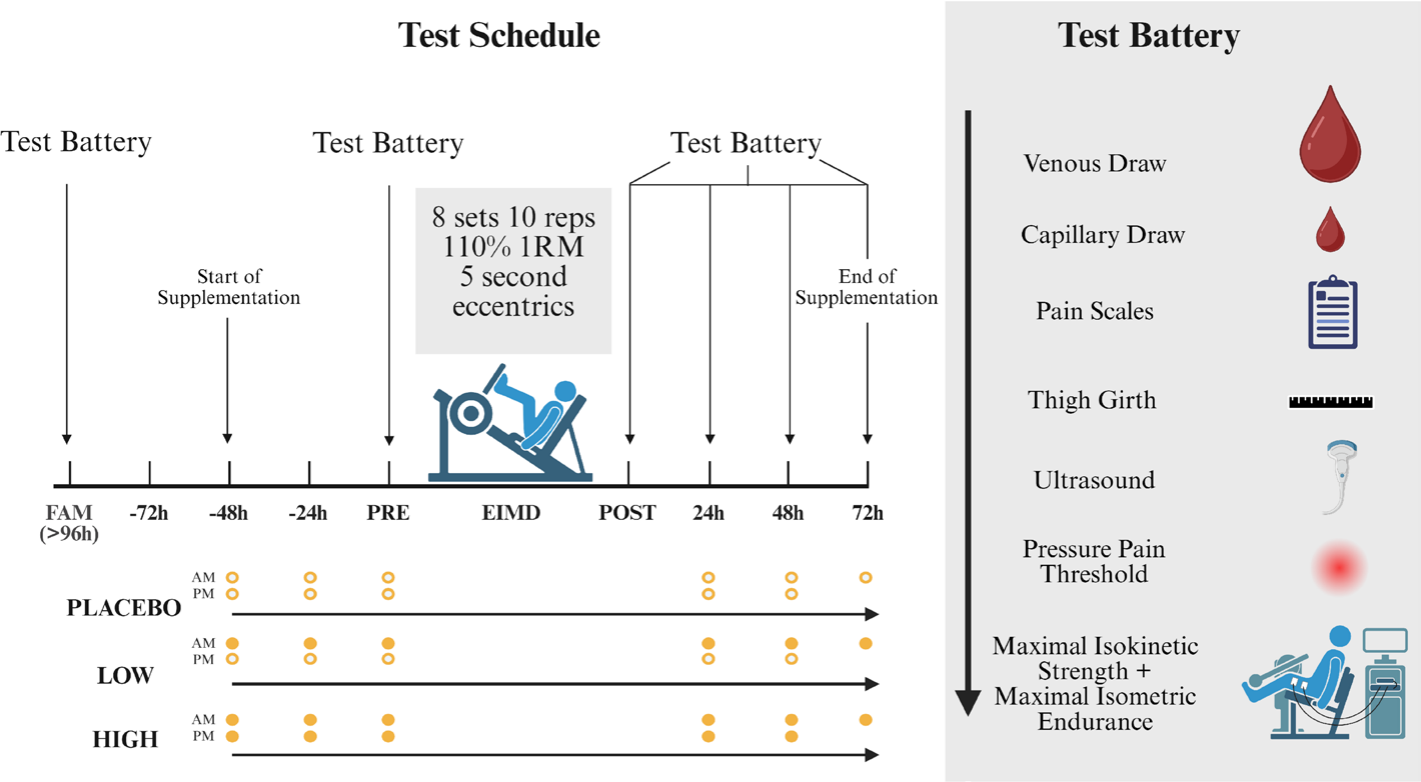
Overview of the experimental design. EIMD, exercise induced muscle damage; FAM, familiarization.

This study employed a double‐blind, randomized, placebo‐controlled, counterbalanced, independent‐groups design. All participants were required to visit the human performance laboratory on five occasions, the first being a familiarization trial at least 96 h before the EIMD protocol (day one). Participants then attended the laboratory on four consecutive days: the first for the EIMD protocol and the following 24‐, 48‐, and 72‐h for post EIMD measurements. Indirect and direct markers of muscle damage and recovery were measured at all time points. The order of tests is outlined in Figure 1. Each visit took place the same time of day (±2 h) to minimize physiological variations due to circadian rhythms (Bumgarner et al., 2021).

### 2.3 Familiarization and 1RM leg press

Approximately 96 h prior to the muscle damage protocol, participants reported to the laboratory where they were familiarized with all tests. After a 5‐min dynamic warm‐up using a self‐selected load on a cycle ergometer (80 revolutions per minute and rating of perceived exertion of 11/20), participants were introduced to the leg press machine. While keeping their heel in contact, participants were instructed to place their feet to the lowest depth on the plate; each rep had a minimal end position of 90° knee flexion. The one repetition max (1RM) predictions were made based on the participant's experience with the leg press and overall resistance background. After a gradual warm‐up at 50%, 70%, and 90% of their predicted 1RM, participants rested for 5 min before attempting their maximal efforts. Increments of 5–10 kg were added after a 5‐min rest between each effort, with the test continuing until failure. After the 1RM was established, participants rested for 2 min before they were familiarized with the timings of the EIMD protocol (5‐s eccentric phase) at 70% of their 1RM for 5 repetitions.

### 2.4 Supplementation

A commercially available hydrolyzed curcumin product was provided by the manufacturer (Zooki, Lancashire, UK). Supplements were packaged in 15 mL sachets, each containing 750 mg of liposomal curcumin (full ingredient list: purified water, lipid curcumin Complex (containing curcumin from turmeric and sunflower oil rich in vitamin E in the form of d‐alpha tocopherol), xylitol, orange seed oil, beta carotene, potassium bicarbonate, oleic acid, and citric acid). The placebo sachets (also produced by the same manufacturer) were made to match for flavor and color (Full ingredient list: purified water, sunflower oil, organic glycerin, xylitol, carrot extract, celery extract, annatto extract, natural citrus oil, potassium bicarbonate, oleic acid, citric acid, vitamin E (d‐alpha tocopherol), and gum acacia). Participants were randomly assigned to one of the three experimental groups: either consuming a double dose of curcumin (HIGH), a single dose of curcumin (LOW) and a placebo (PLA), or two doses of a placebo using a counterbalanced number generator created in Excel (Figure 1). Supplementation began 2 days prior to the EIMD protocol, with one sachet ingested every 12 h (8 am and 8 pm) for 7 days. The participants reported no adverse gastrointestinal effects. Self‐reported participant compliance was 100%.

### 2.5 Exercise‐induced muscle damage (EIMD) protocol

Prior to the EIMD protocol, the identical warm up from familiarization was followed. Participants then completed 8 sets of 10 reps at 110% of their 1RM, incorporating a 5‐s (metronome‐assisted) eccentric phase with each rep, with an assisted concentric return phase (two spotters). A 5‐min seated rest was prescribed between each set. For further details of the EIMD protocol, see McFarlin et al. (2016). Two additional sets were completed in this study compared to McFarlin et al. (2016) to ensure sufficient EIMD.

### 2.6 Blood sampling

Upon arrival in the laboratory each morning, venous samples were drawn from the antecubital vein into EDTA‐Na^2^ (6 mg) vacutainers and immediately spun at 15,000 rpm for 15 min at 4°C. Plasma supernatant was then separated and stored at −80°C. Once all samples had been collected, they were transferred to an external lab provider (Affinity Biomarker Labs, London) for analysis of the following biomarkers: Creatine Kinase (CK), selected interleukins (IL‐1B, IL‐6, IL‐4, IL‐10, and IL‐1RA) and Tumor Necrosis Factor‐alpha (TNF‐α). Table 2 illustrates the detectable range and performance of each assay. To estimate redox status, antioxidant capacity and reactive oxygen species activity (hydroperoxides) were assessed using the Free Oxygen Radicals Defence Test (FORD; Cat. No. AD‐12136) and the Free Oxygen Radicals Test (FORT; Cat. No. AD‐12107‐A), both from Callegari SpA (Catellani Group, Parma, Italy). Prior to all blood sampling, the participants were seated at rest for 10 min. Capillary blood samples (50 μL for FORD and 20 μL for FORT) were taken from the earlobe, processed, and analyzed immediately at room temperature according to the manufacturer's instructions. Briefly, capillary samples were mixed with the reagent, centrifuged, and analyzed colorimetrically (CR3000, Callegari SpA, Catellani Group, Parma, Italy). The ratio (FORT:FORD) was used as an index of oxidative stress (OSI, oxidative stress index). See Lewis et al. (2016) for a detailed description of the FORD/FORT assays.

**TABLE 2:** The detectable range of the ELISA panels analyzed by Affinity Biomarkers Ltd. assays and assays from Callegari SpA.

Marker	Detectable range	Intra‐assay coefficient (%)
Creatine Kinase	1.0–7800 U/L	0.9
IL‐1B	0.27–576 ng/L	3.8
IL‐4	0.05–208 ng/L	5.8
IL‐6	0.11–1470 ng/L	4.9
IL‐10	0.015–367 ng/L	3.1
TNF‐α	0.13–347 ng/L	2.8
IL‐1RA	6.3–2000 ng/L	5.3
FORT	1.22–4.56 mmol/L H_2_O_2_	3.9
FORD	0.25–3 mmol/L Trolox	3.7

### 2.7 Subjective muscle soreness

Perceived levels of muscle soreness were assessed using both the visual analog scale (VAS) and the Borg CR‐10 scale (BORG). Participants stood with their feet shoulder‐width apart and hands on their hips. They were instructed to perform at least two bodyweight squats before completing the pain scale assessments. VAS soreness was assessed by drawing a vertical line perpendicular to the continuum line (0 mm = no soreness; 200 mm = extreme soreness) (Bijur et al., 2001). The Borg CR‐10 scale was completed by circling the number (0 = nothing at all; 10 = extremely strong) that correlated best with their perceived pain (Borg, 1998).

### 2.8 Objective muscle soreness

Pressure pain threshold (PPT) was used to gauge objective muscle soreness in the rectus femoris muscle of the dominant leg. All PPT measurements were taken with participants lying supine. The PPT site was located two‐thirds of the way down from the anterior superior iliac spine to the superior border of the patella, with the rectus femoris muscle belly palpated and marked. PPTs were assessed using a handheld mechanical pressure algometer (FPX, Wagner Instruments, Connecticut, USA) at a pressure rate of ~1 kg force per second (kgf/s) over a stimulation area of 1 cm^2^ (Sylwander et al., 2021; Vaegter et al., 2019). Participants gave the verbal instruction “there” when the pressure was first perceived as painful, and PPT was quantified as the kgf applied at that point. Three PPT assessments were completed, each separated by 1‐min rest, and the mean was utilized for analysis. During the pilot study, PPTs and ultrasounds were assessed three times at 10‐min intervals to estimate intra‐rater reliability among 13 participants using intraclass correlation coefficients (ICC). Minimum detectable change (MDC) and standard error of measurement (SEM) were calculated (Table 3). Pilot testing revealed that Researcher A (who performed 98% of the pressure pain threshold [PPT] readings) had an average coefficient of variation (CV) of 3.76%, while Researcher B demonstrated an average CV of 4.08%. To ensure consistency and reduce measurement variability, the same researcher assessed each individual across all trials. This approach was employed to prevent inter‐rater variability from confounding the results and to strengthen the internal validity of the data.

**TABLE 3:** Reliability testing for PPT and ultrasound measures.

Method	Value	ICC	SEM	MDC
PPT (*n* = 11)	5.21 ± 0.21	0.99	0.19	0.52
Ultrasound (*n* = 13)	23.81 ± 0.77	0.99	0.54	1.51

*Note*: Means ± SD.

Abbreviations: ICC, intraclass correlation coefficient; MDC, minimal detectable change; PPT, pressure pain threshold; SEM, standard error of measurement.

### 2.9 Ultrasound assessment

Muscle swelling was assessed using a 2D ultrasound scanning device (LogicScan 128 EXT‐1Z, Telemed). While supine, the ultrasound assessment site was located and marked on the vastus lateralis of the dominant leg. To locate the site, muscle girth (cm) was first measured across the PPT site of the rectus femoris, and then 10% of the circumference was calculated distal to the PPT marker (Pasta et al., 2010). All images were taken at a depth of 70 mm, with the mean of three images used for analysis. The distance (mm) between the superior aponeurosis and inferior aponeurosis was calculated using ImageJ (US National Institutes of Health, Bethesda, Maryland, USA).

### 2.10 Muscle function

Maximal isokinetic strength (peak torque) of the quadriceps was measured using the Cybex Norm Isokinetic System (Computer Sports Medicine Inc., USA) at an angular velocity of 60°/s through full knee extension. Once in the starting position with the leg flexed at a 90° angle, participants forcefully extended their leg against resistance through the full range of motion for three continuous maximal repetitions per set, completing a total of three sets with a 1‐min rest between each set. The highest recorded value was used for analysis.

Maximal isometric endurance for the knee extensors was tested at a 45° angle with the shin pad positioned 2 cm above the lateral malleolus of the fibula, attached to a load cell. Muscle endurance was measured while holding an isometric contraction at 50% of their peak torque for as long as possible. The time was calculated from the beginning of force production to the point where the value dropped below the 10% threshold of the target (50% MVC) for 3 s or longer (Zech et al., 2008).

### 2.11 Statistical analysis

The primary outcome measure of mechanical PPT was used to calculate the required sample size a priori using G*Power (Faul et al., 2007). With an expected effect size of *d* = 0.42, to achieve a power of 80% at an alpha level of 0.05 with a two‐way (3 × 5) repeated measures ANOVA, a total of 36 participants were required. Prior to formal statistical testing, data were checked for normality. Statistical analysis was performed using IBM SPSS Statistics Version 26.0 (IBM Corp., Chicago, IL). A two‐way mixed measures ANOVA was used to investigate possible differences in absolute and relative changes (%) in biomarkers and performance indicators between the groups (PLA, LOW, and HIGH) and within groups across time points (Pre‐EIMD, Post‐EIMD, 24‐, 48‐, and 72‐h post muscle damage). Assumptions of sphericity were assessed using Mauchly's test, with any violations adjusted using the Greenhouse–Geisser correction. Statistical significance was set at *p* < 0.05. For any statistically significant two‐way interaction, post hoc analysis was performed with Bonferroni correction. All data are reported as the mean ± standard deviation (SD) with 95% confidence intervals (CIs) unless otherwise indicated.

## 3 RESULTS

### 3.1 Subjective muscle soreness

**FIGURE 2:**
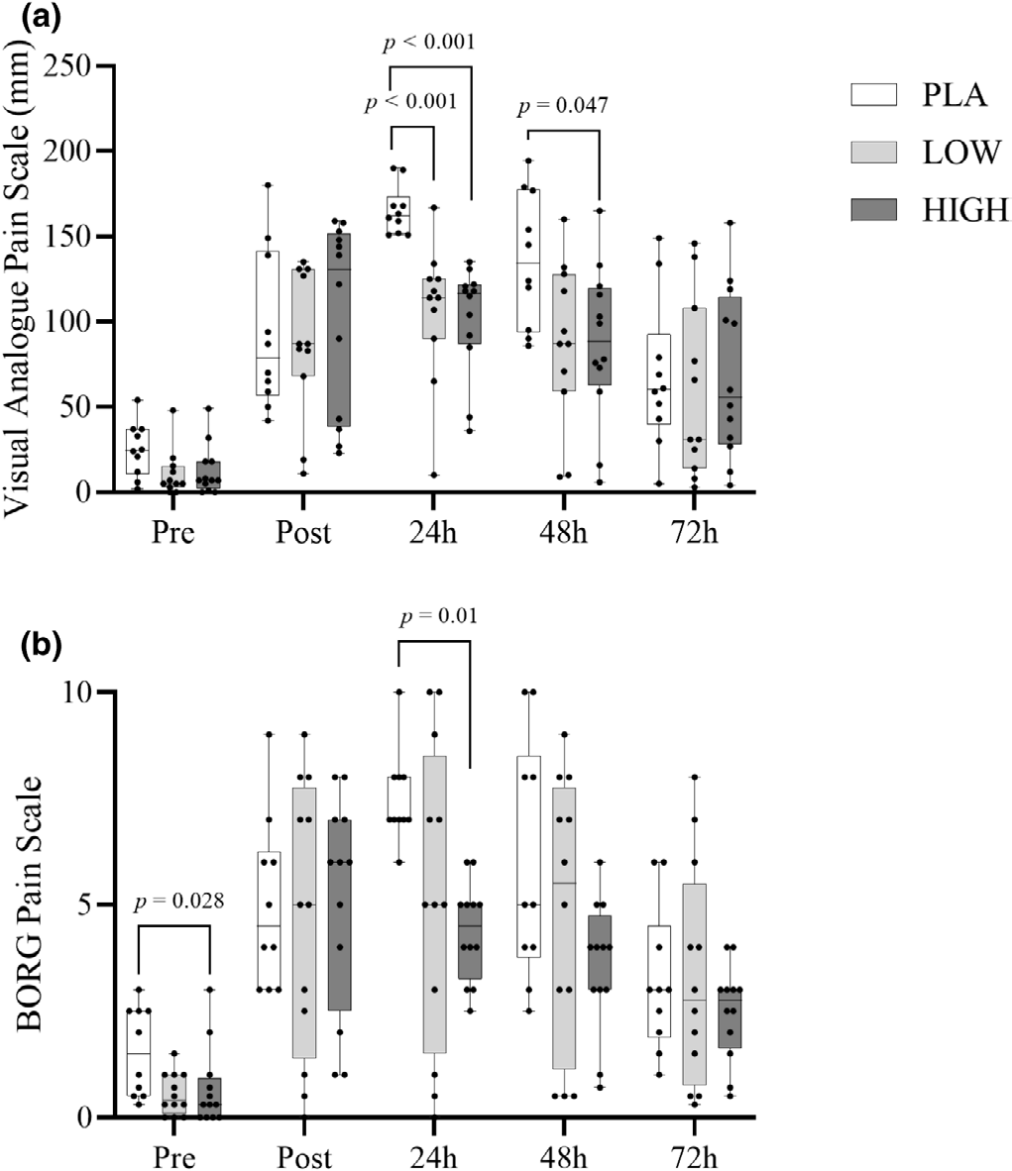
Subjective pain responses assessed using the VAS Pain Scale (a) and BORG Pain Scale (b) before, immediately after, and during recovery from a bout of muscle‐damaging exercise. Data are presented as box and whisker plots showing the median, interquartile range (IQR), and range.

A significant time effect was observed for the VAS (*F*_3,95.15_ = 72.68, *p* < 0.001) and BORG (*F*_2.88,89.45_ = 54.45, *p* < 0.001) scales, suggesting there were changes in muscle soreness over time induced by the EIMD. Further post hoc Bonferroni tests indicated differences from baseline at all time points in lower‐limb muscle soreness (VAS and BORG both *p* < 0.05). No group effects were seen in VAS (*F*_2,31_ = 2.08, *p* > 0.05) or BORG (*F*_2,31_ = 1.91, *p* > 0.05). However, Time*Group interaction effects were observed for both the VAS (*F*_6.1,96.15_ = 3.64, *p* < 0.05) and BORG scales (*F*_5.7,124_ = 0.77, *p <* 0.05). Subjective muscle soreness assessed by VAS was lower in LOW and HIGH compared to PLA (*p* < 0.05) 24 h after EIMD but was not different between LOW and HIGH. At 48 h post EIMD, HIGH still displayed significantly lower soreness than PLA, indicating there may be a dose–response between LOW and HIGH (Figure 2). BORG also showed differences (*p* < 0.05) between groups at Pre, with PLA being higher than LOW, and at 24 h post‐exercise HIGH being significantly lower than PLA.

### 3.2 Pro‐ and antioxidant status

**FIGURE 3:**
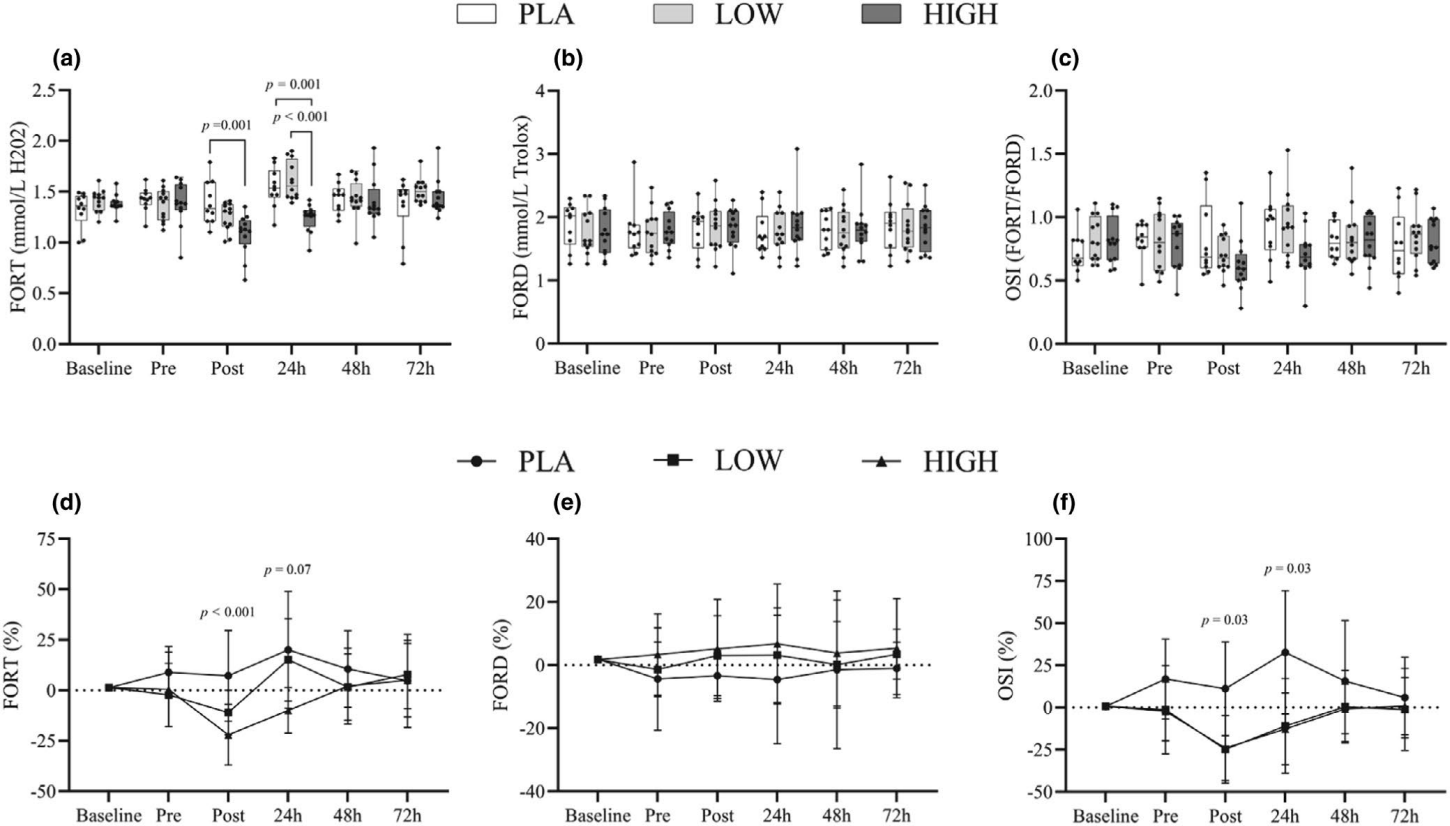
Box‐and‐whisker plots (a–c) display absolute whole blood concentrations of FORD (a), FORT (b), and OSI (c) measured at three time points: pre‐exercise (baseline), immediately post‐exercise, and during recovery following muscle‐damaging exercise. Each plot shows the median, interquartile range (IQR), and full range. Line graphs (d–f), located below the box plots, present the mean ± standard deviation (SD) of the relative change from baseline for FORD (d), FORT (e), and OSI (f), aligned with the same time points.

FORT analysis showed significant main effects for Time (*F*_5,155_ = 8.24, *p* < 0.05), Group (*F*_2,31_ = 4.17, *p* < 0.05), and Time*Group (*F*_10,155_ = 4.4, *p* < 0.05). Post hoc tests indicated differences (*p* < 0.05) from baseline to immediately post intervention and at 24‐, 48‐, and 72‐h post muscle damage, reflecting shifts in hydroperoxide levels during recovery. Specifically, the HIGH group displayed a lower FORT than PLA immediately post EIMD (−23%) and 24 h post‐exercise (*p* < 0.05) compared to both PLA and LOW (−19.5%). When expressed as percentage change from baseline, the oxidative stress index was lower in both the LOW and HIGH groups compared to PLA immediately and 24 h post EIMD, as shown in Figure 3. No significant differences were observed in FORD.

### 3.3 Inflammation and muscle damage

**FIGURE 4:**
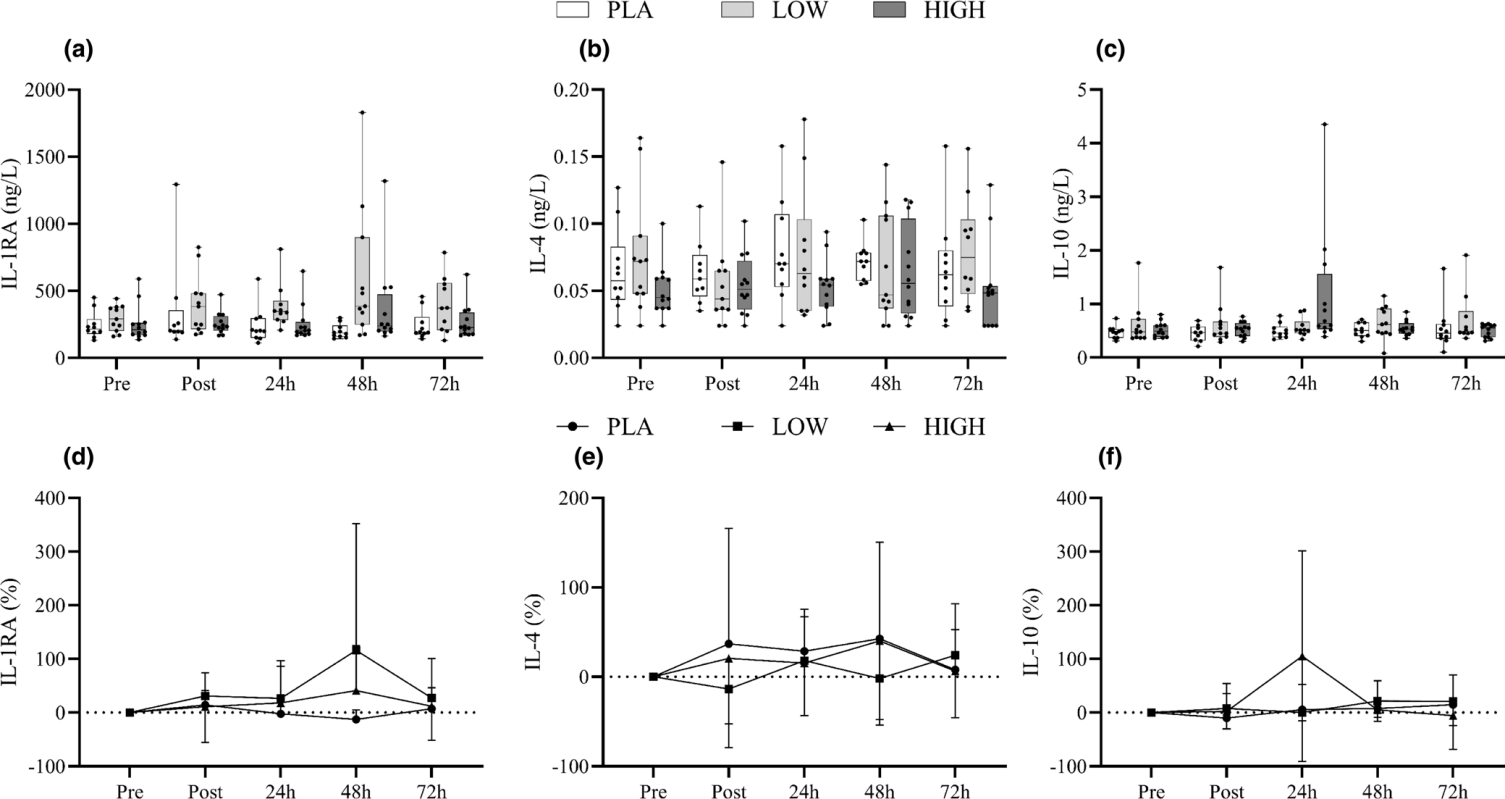
Box‐and‐whisker plots (a–c) show plasma concentrations of the anti‐inflammatory cytokines IL‐1RA (a), IL‐4 (b), and IL‐10 (c), measured at pre‐exercise (baseline), immediately post‐exercise, and during the recovery period across multiple days. Each plot displays the median, interquartile range (IQR), and full range of values. Line graphs (d–f), positioned below the box plots, present mean ± standard deviation (SD) of the relative change from baseline for IL‐1RA (d), IL‐4 (e), and IL‐10 (f), aligned with the same time points.

**FIGURE 5:**
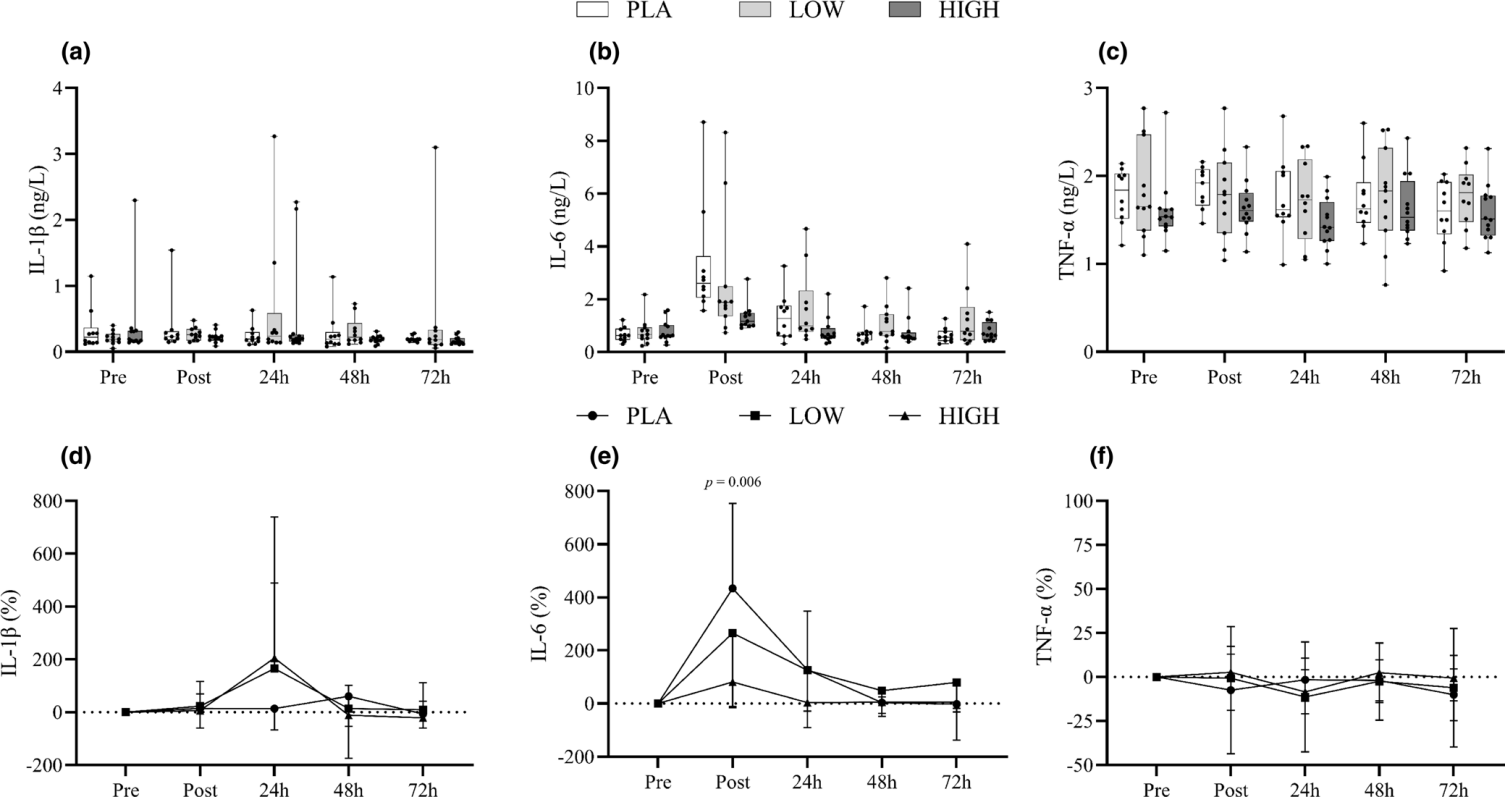
Box‐and‐whisker plots (a–c) display absolute plasma concentrations of the pro‐inflammatory cytokines IL‐1β (a), IL‐6 (b), and TNF‐α (c), measured pre‐exercise (baseline), immediately post‐exercise, and during the recovery period following muscle‐damaging exercise. Each plot shows the median, interquartile range (IQR), and full range of values. Line graphs (d–f), located below the box plots, present mean ± standard deviation (SD) of the relative change from baseline for IL‐1β (d), IL‐6 (e), and TNF‐α (f), using the same time points.

**FIGURE 6:**
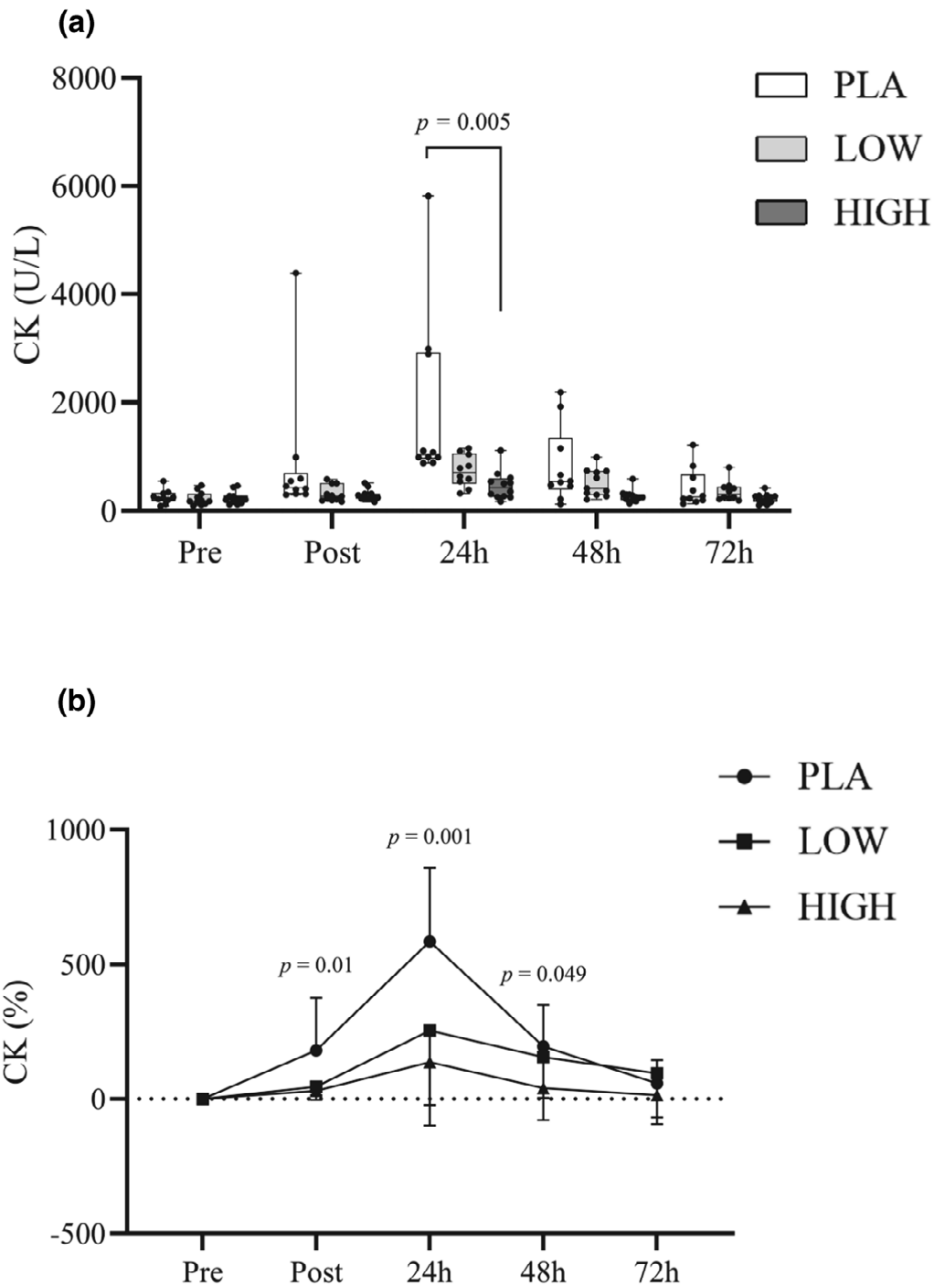
Box‐and‐whisker plot (a) shows absolute plasma concentrations of creatine kinase (CK), a marker of muscle damage, measured pre‐exercise (baseline), immediately post‐exercise, and during the recovery period following muscle‐damaging exercise. The plot displays the median, interquartile range (IQR), and full range of values. Line graph (b), shown below the box plot, presents the mean ± standard deviation (SD) of the relative change from baseline, aligned with the same time points.

IL‐6 both absolute and relative changes (from PRE) revealed Time (*F*_4,112_ = 20.76, *p <* 0.05) and Time*Group (*F*_8,112_ = 2.36, *p <* 0.05) interactions. Post hoc analysis found differences between pre and post, as well as pre and 24 h (see Figure 5). Differences were also seen between post and 24 h, 48 h, and 72 h. Peak IL‐6 concentrations were seen directly post EIMD. Post hoc analysis could not identify between‐group differences in absolute terms; however, the increase in IL‐6 (%) was greater in PLA compared to HIGH immediately post‐exercise (*p* < 0.05). CK showed effects across Time (*F*_4,116_ = 18.82, *p <* 0.05), Group (*F*_2,29_ = 4.2, *p <* 0.05), and Time*Group (*F*_8,116_ = 4.86, *p <* 0.05). Further analysis showed differences between pre and 24 h (*p <* 0.05) and pre and 48 h (*p <* 0.05). Further time point differences were seen between post and 24 h (*p <* 0.05) and 48 h, 24 h, and 72 h (*p <* 0.05). At 24 h post muscle damage, differences in CK existed (*p <* 0.05) between PLA and HIGH, with HIGH displaying CK −75% lower than PLA. Compared to Pre, however, changes in CK levels between groups were observed immediately post‐exercise, as well as at 24 h and 48 h, with the HIGH group demonstrating the smallest deviation from Pre. Other than IL‐6 and CK, no Time*Group or between‐group changes were found in the biomarkers in Figures 4, 5, and 6. Time differences (*p* < 0.05) were found in swelling measures; see Table 4.

**TABLE 4:** Changes in quadriceps swelling post EIMD across recovery days.

Measure	Group	Pre	Post	24 h	48 h	72 h
Thigh girth (cm)	PLA	55.43 ± 5.01	**56.13 ± 4.48**	**56.31 ± 4.07**	56.03 ± 5.05	55.8 ± 5.03
	LOW	55.63 ± 3.79	56.01 ± 4.22	**56.45 ± 3.93**	56.72 ± 4.63	55.91 ± 3.98
	HIGH	54.03 ± 3.86	**55.02 ± 4.55**	54.73 ± 4.25	54.63 ± 4.14	54.38 ± 4.14
VL ultrasound (mm)	PLA	26.6 ± 4.88	**28 ± 5.49**	27.4 ± 5.32	27.8 ± 4.98	27.6 ± 5.11
	LOW	27.1 ± 5.9	**28.9 ± 5.9**	**28 ± 5.4**	**27.7 ± 5.4**	28 ± 5.6
	HIGH	25 ± 2.4	25.9 ± 3	25.1 ± 2.7	25.6 ± 2.8	25.7 ± 2.6

*Note*: Data are represented as mean ± SD. Bold, *p* < 0.05 versus Pre.

Abbreviation: VL, vastus lateralis.

### 3.4 Pain pressure threshold

**FIGURE 7:**
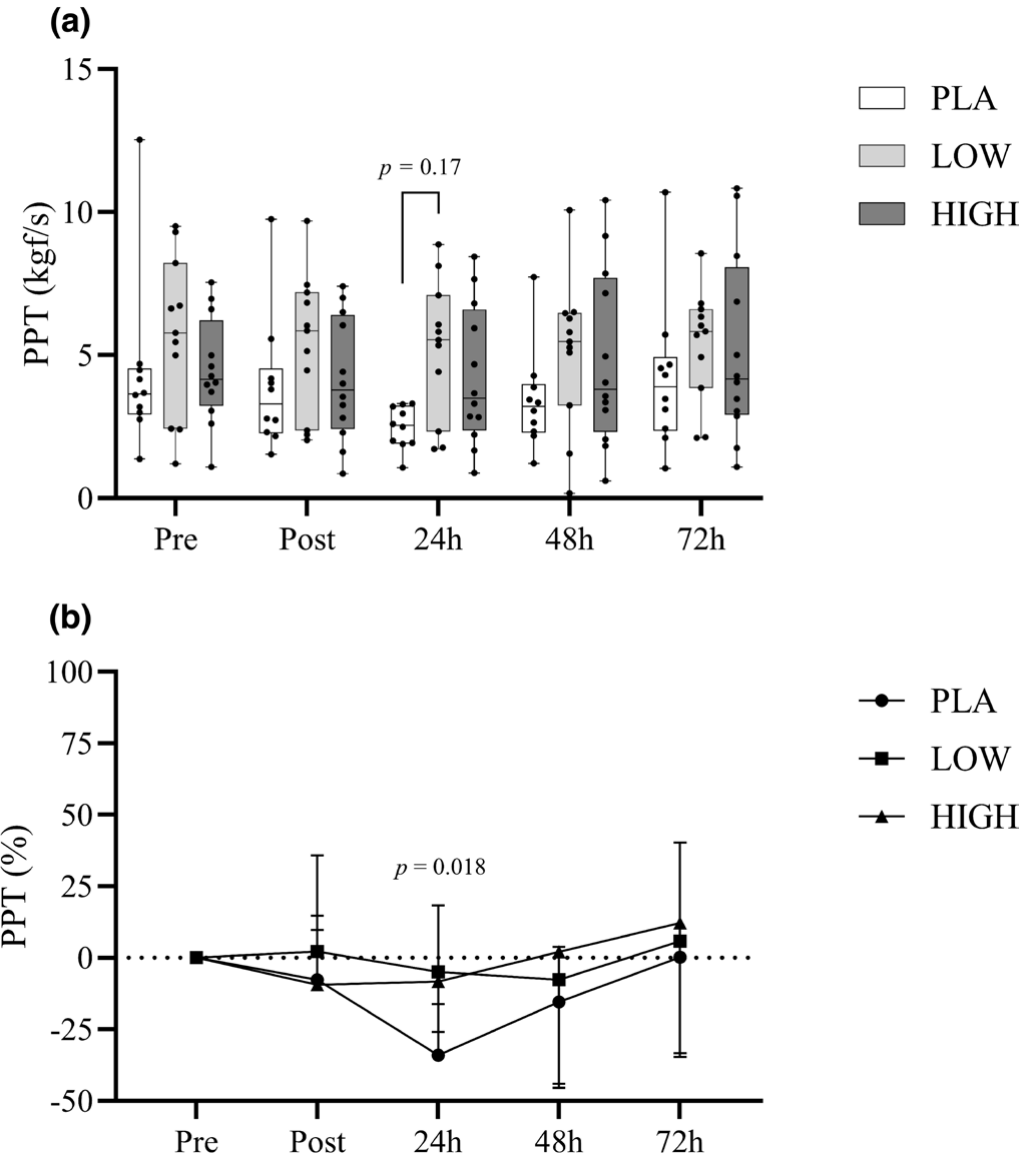
Box‐and‐whisker plot (a) displays pain pressure threshold (PPT) values measured at baseline (pre‐exercise), immediately post‐exercise, and throughout the recovery period to assess muscle sensitivity. The plot presents the median, interquartile range (IQR), and full range of values. Line graph (b), located below the box plot, shows the mean ± standard deviation (SD) of the relative change from baseline, aligned with the same time points.

Both absolute and relative changes in PPT (from PRE) revealed time (*F*_4,120_ = 4.672, *p* < 0.05) interactions. Compared with pre‐exercise values, PPTs reduced post‐exercise. Post hoc analyses of absolute PPT values revealed significantly lower PPT measurements (*p* < 0.05) 24 h after EIMD. Similarly, post hoc observations of relative PPT indicated significantly reduced (*p* < 0.05) values in the PLA condition compared to both the LOW and HIGH conditions at the same time point. However, no group or Time*Group interactions were observed. PPT relative to Pre (%) showed group differences at 24 h between PLA and both LOW and HIGH, with PLA exhibiting the greatest deviation from Pre (see Figure 7).

### 3.5 Muscle performance

**FIGURE 8:**
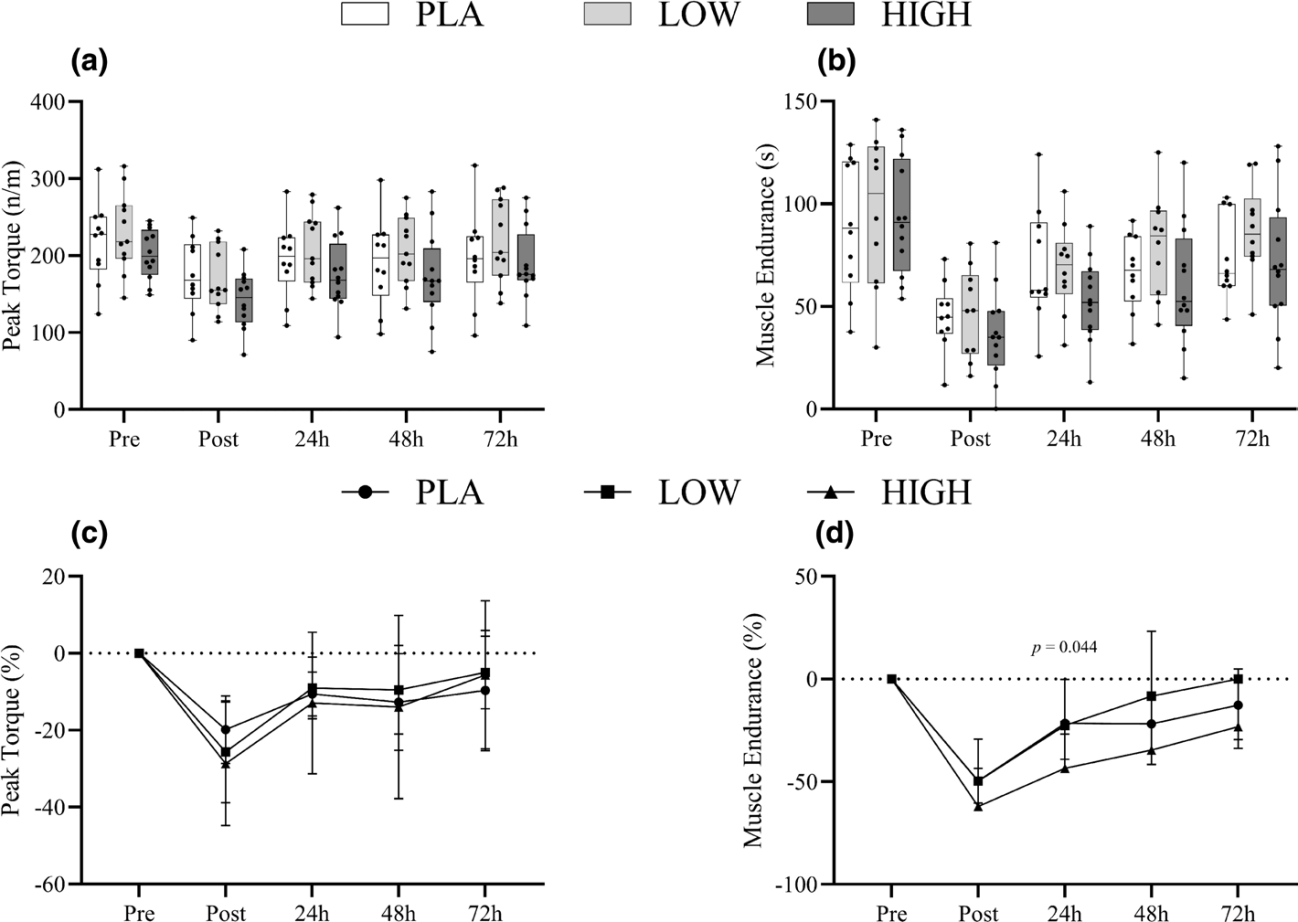
Box‐and‐whisker plots (a, b) show muscle function outcomes, including peak torque (a) and isometric endurance (b), measured pre‐exercise (baseline), immediately post‐exercise, and during the recovery period following muscle‐damaging exercise. Each plot presents the median, interquartile range (IQR), and full range of values. Line graphs (c, d), located below the box plots, display the mean ± standard deviation (SD) of the relative change from baseline for peak torque (c) and isometric endurance (d), aligned with the same time points.

Peak torque revealed a Time effect (*F*_2.24,69.34_ = 38.89, *p >* 0.05) but not Time*Group (*F*_4.47,69.34_ = 0.727, *p >* 0.05) or Group interactions (*F*_2,31_ = 1.61, *p >* 0.05), no differences were observed in relative terms between Groups (%). Muscle endurance also revealed Time (*F*_2.23,64.7_ = 55.56, *p >* 0.05) but not Time*Group (*F*_4.47,64.7_ = 1.316, *p >* 0.05) or Group interactions (*F*_2,29_ = 0.88, *p >* 0.05). However, differences were observed in relative terms 24 h post EIMD, with HIGH showing significantly greater deviation from Pre values than PLA and LOW (Figure 8).

## 4 DISCUSSION

The aim of this study was to investigate the effect of two different doses (750 mg/day and 1500 mg/day) of a hydrolyzed curcumin supplement on physiological recovery following EIMD. To the author's knowledge, the present study was the first to examine the effect of hydrolyzed curcumin on EIMD recovery. The key finding was that HIGH (1500 mg/day) exhibited superior effects in mitigating hydroperoxides, IL‐6, and CK release post‐exercise, compared to LOW (750 mg/day) (see Figures 3 and 4). Additionally, both curcumin groups demonstrated significant reductions in subjective pain measures (VAS) and changes in pain pressure thresholds during the recovery days (see Figure 5). Despite alterations in pain levels and inflammation markers, the curcumin groups did not demonstrate a significant advantage in muscle performance recovery. These findings support previous research indicating that curcumin supplementation may modulate some aspects of recovery (Basham et al., 2020; Drobnic et al., 2014; McFarlin et al., 2016; Nicol et al., 2015).

This is the first study to evaluate the effects of curcumin on recovery, utilizing the FORD and FORT assays to assess antioxidant capacity and hydroperoxide levels. The present study found that FORT levels in the HIGH group were 23% lower than in the PLA group immediately after exercise and 19.5% lower 24 h post‐exercise. Curcumin is thought to both upregulate nuclear factor erythroid 2 related factor 2 and downregulate the oxidative stress expression of nuclear factor kappa‐B (NF‐kB) (37). Although FORT levels were significantly reduced in the HIGH group, no differences were observed in FORD between groups. However, both the curcumin groups also reduced oxidative stress as estimated using the ratio of FORT:FORD. This contrasts with the findings of Takahashi et al. (2014), who reported that curcumin reduced oxidative stress after EIMD by enhancing antioxidant capacity (biological antioxidant potential and glutathione). However, in the present study, no changes were observed in antioxidant capacity, but rather in hydroperoxide levels, a downstream marker of ROS. In the present study, a 48‐h loading phase was employed. However, this duration might have been too brief to significantly boost plasma antioxidant capacity, as longer loading phases, such as 28 days, have been demonstrated to effectively increase antioxidant levels (Basham et al., 2020). Interestingly, Takahashi et al. (2014) reported differences in antioxidant levels with supplementation taken just 2 h before and after exercise. The reduction in FORT levels observed supports the evidence that curcumin decreases the oxidative stress properties of NF‐kB as seen by Sahin et al. (2016).

The observed blunting of the CK rise directly post EIMD and through to 48 h post exercise in HIGH further supports the notion that consumption of curcumin close to exercise (<2 h) downregulates the cyclooxygenase (COX‐2) pathway. This could influence cell permeability, offering a protective effect by altering membrane structure and improving its integrity (Nanavati et al., 2022). These findings agree with Tanabe et al. (2019) who found taking curcumin after exercise reduced CK levels across recovery days. Downregulating COX‐2 pathway signaling also reduces prostaglandin production, which is thought to reduce inflammation as well as pain severity (Hatcher et al., 2008; Nanavati et al., 2022). A reduction in subjective pain (VAS and BORG) was observed at both 24‐ and 48‐h post‐exercise with both LOW and HIGH protocols. PPT markers were significantly reduced 24 h post‐exercise; however, this reduction was attenuated in both curcumin groups. The observed reduction in pain may be associated with the 75% decrease in CK levels following exercise, indicating less initial muscle damage and potentially a reduction in subsequent muscle damage in the HIGH group. Additionally, this effect could be attributed to the significant decrease in the inflammatory cytokine IL‐6 observed in the HIGH group, as lower IL‐6 levels may reflect a dampened inflammatory response. This reduction in inflammation could contribute to decreased muscle soreness and pain, as IL‐6 is known to play a key role in promoting inflammation and sensitizing pain receptors in response to muscle injury. Consequently, the lowered IL‐6 levels in the HIGH group may help mitigate both the perception of pain and the progression of inflammation induced secondary muscle damage (Sebba, 2021). This may support the notion that prostaglandin production was inhibited, as both curcumin groups exhibited reduced perceived muscle soreness. These findings support the work from Nicol et al. (2015) who showed decreases in perceived muscle soreness alongside decreases in CK as well as no changes in TNF‐α while taking a high curcumin dose of 2500 mg/day. Thus, perhaps suggesting that curcumin may act through multiple biochemical pathways rather than just simply suppression of the COX‐2 pathway and may be more enzyme/tissue specific (Basham et al., 2020).

Although a decline in perceived pain was observed throughout the recovery period from both curcumin groups, the present study did not discern variations in inflammatory and anti‐inflammatory markers among groups other than IL‐6. Curcumin is recognized for mitigating inflammation by suppressing the prominent COX‐2 pathway, resulting in a reduction of inflammatory markers like TNF‐α and IL‐4 (Fernández‐Lázaro et al., 2020). Yet, the current study revealed no distinctions in inflammatory and anti‐inflammatory markers, or swelling, with the exception of IL‐6. In agreement with our findings, Sciberras et al. (2015), Basham et al. (2020), and Drobnic et al. (2014) have shown that while inflammation biomarkers tend to be reduced post‐exercise with curcumin, significant reductions are lacking in the literature comparing low and high doses. Therefore, the reductions in muscle soreness observed may be attributed to mechanisms beyond the COX‐2 pathway, such as the NF‐kB pathway, which has been associated with pain modulation (Ahmed et al., 2019). Additionally, the significant decrease in CK levels observed post‐exercise in the HIGH group suggests less muscle damage, further contributing to the reduced soreness (Figures 7 and 8).

Loss of muscle strength is a frequently utilized marker for quantifying muscle damage and assessing recovery following exercise‐induced muscle damage (Shoji et al., 2021). In this study, muscle function declined following the damaging protocol, as evidenced by both muscle performance tests, peak torque and isometric endurance. While no differences were observed in peak torque, a significant reduction in muscle endurance at 24 h post‐EIMD was noted between the LOW and HIGH groups, with the HIGH group showing markedly delayed recovery (*p* < 0.05). Contrary to previous studies where curcumin has either shown no significant differences in MVC peak torque after 7 days of supplementation (Tanabe et al., 2019) or attenuated reductions in peak torque compared to placebo after 8 weeks of supplementation (Jäger et al., 2019). In another randomized controlled trial investigating antioxidant supplementation with N‐acetylcysteine (Michailidis et al., 2013), similar results to the present study were reported, with improvements in markers of inflammation, muscle damage, and oxidative stress with antioxidant supplementation; however, these benefits were accompanied by a blunting of the muscle repair process following eccentric exercise. Given the LOW dose of curcumin did not impair muscle recovery in the present study, it is possible that an “inverted U” dose relationship exists whereby high doses above a certain threshold may actually impair recovery compared to placebo. This result warrants further investigation to determine whether it represents a singular occurrence or a consistent pattern.

There are limitations in the current study. The severity of the EIMD may have created conditions too extreme for the dosage of curcumin/polyphenols to show any discernible effect across some of the inflammatory/anti‐inflammatory markers. Secondly, the present study did not control the diet of the participants or implement a food diary. This was decided to reduce the burden on the participants who agreed to participate. Prior research has shown that an elevated dietary consumption of fruits and vegetables correlates with diminished concentrations of systemic inflammatory markers and augments levels of anti‐inflammatory cytokines (Dryer‐Beers et al., 2024). Consequently, including participants with varying levels of fruit and vegetable intake may have introduced confounding factors affecting inflammatory and anti‐inflammatory markers. However, this also provided real‐world insight, as hydrolyzed curcumin demonstrated recovery effects within a typical population, thus enhancing the study's ecological validity.

In conclusion, both curcumin groups (750 mg/day and 1500 mg/day) improved recovery post EIMD through reduced perceived pain, oxidative stress, and muscle damage markers, demonstrating that hydrolyzed curcumin may be effective in modifying recovery. Moreover, a higher dose of 1500 mg/day showed greater effects in reducing VAS, CK, and FORT compared to a lower dose. Nevertheless, although these effects were substantial, they did not confer performance benefits in the HIGH group and, in fact, impaired recovery as measured by the muscle endurance test. These results support previous evidence that the bioavailability of curcumin is low, and a high dose is needed to elicit effects.

## AUTHOR CONTRIBUTIONS

T.A.H., S.C., I.G., N.A.L., J.H., C.R.P., and L.H. conceived and designed research; T.A.H. and S.C. performed experiments; T.A.H. and L.H. analyzed data and interpreted results of experiments; T.A.H. prepared figures and drafted manuscript; T.A.H., S.C., N.A.L., J.H., I.G., C.R.P., and L.H. edited and revised manuscript; T.A.H., S.C., N.A.L., J.H., I.G., C.R.P., and L.H. approved final version of manuscript.

## FUNDING INFORMATION

This research project was funded by a grant provided by Zooki, who had no involvement in the study design or preparation of this manuscript.

## CONFLICT OF INTEREST STATEMENT

The authors state that there are no conflicts of interest regarding the publication of this research.

## Data Availability

The data that support the findings of this study are available from the corresponding author T.A. Helder, upon reasonable request.
